# Supplementary material for: COVID-19 vaccine hesitancy in Malaysia: Exploring factors and identifying highly vulnerable groups
Source: PLoS One. 2022 Jul 8;17(7):e0270868. doi: 10.1371/journal.pone.0270868 (PMC9269452; doi:10.1371/journal.pone.0270868)
Supplement: Attachment — Submitted filename: Response to Reviewers.doc [file pone.0270868.s002.doc]

**Author Responses based on Editor and Reviewers comments:**

We would like to thank both reviewers and editors for their constructive comments and helpful suggestions to improve this article. All amendments are highlighted in trackchanges and more specific responses in the following table.

| **Review** | **Comments** | **Responses** |
| --- | --- | --- |
| **R1** | Reviewer #1: This article is interesting and very timely. The author has presented the paper well in accordance with scientific rules. The findings of this study are also very important for the government, especially to speed up the slow vaccination process. On the other hand, resistance to vaccination appears for various reasons. Whether it's related to insufficient information dissemination, inappropriate policies, or even the spread of hoax news related to the COVID-19 vaccine. This is actually the trigger for why the vaccination did not work as expected. The triggers for this slow vaccination process have not been seen in this paper. In fact, it is very important to know as well as an academic footing on the significance of this research. Therefore, the author is recommended to add a specific sub-section that discusses the factors of success and inhibition of vaccination which has been done by previous authors during the COVID-19 pandemic.. | Thanks for the comments and suggestions. Reviews of previous studies on the phenomenal factors of vaccine hesitancy have been included in the introductory section as suggested by the reviewer. |
| **R1** | In the introduction, the author also needs to briefly explain what community vulnerability is so that the general readers are able to grasp the theoretical meaning of this paper. This gap has implications for the discussion section where the author actually wants to refer to the previous literature but the literature review section does not yet exist. Should all of these issues be addressed in revision, I would recommend that the essay be published | Thanks for the comments and suggestions. Discussion of vulnerabilities and highlights of previous studies have been included in the introductory section as suggested by the reviewers. The gaps in this study have also been discussed in the conclusions section. |
| **R2** | Topic - It's too long and needs to revise accurately. | We have revised it accordingly. |
| **R2** | Abstract -The author needs to highlight the novelty and implication of the study properly | The novelty of this study was included in the abstract section, as suggested by the reviewer. The implications of the study have already been discussed in the abstract section. |
| **R2** | Introduction - Overall good and very clear. - The statement of problem has been well written and straightforward. But, are the issues presented accurately in describing the current situation in 2022? | Thank you. Based on the reviewer comments, the document has been updated to include the most recent statistical data (year 2022) on vaccination participation in Malaysia and Sabah in particular. |
| **R2** | Literature Review - Too general and only summarized in the introduction. | Literature reviews are deliberately placed in the introductory section to follow the standard of this journal format.  Several literature reviewers discussing the key issues in this study have also been added in the introductory section. |
| **R2** | Literature Review  - The literature review should improve by referring to the latest studies on the subject matter, especially in 2022. | Thank you. Reviews of the most recent studies (year 2022) have already been incorporated in this paper, as requested by the reviewer. |
| **R2** | Conclusion  - The authors conclude all data underlying the findings in the manuscript but still need to highlight the novelty and implication of the study correctly.  - Need to provide suggestions from the study. | We have amended based on reviewer’s comments. We also have included the novelty & recommendations of this study. |
| **Editor** | While the content of your paper is valuable and addressing an interesting and important topic, the research lacks clarity on some parts of methodology and the analysis of data.  The sampling distribution have significant demographic variations in the number of registrations and could influence the results of the hypothesis**.** the author needs to discuss the variations in the sample and their relation to the hypothesis. | Let's look at Table 4, especially for Cluster 2. There is a difference in the percentage value between respondents who have registered for vaccination (37.3%) and those who have not registered (62.7%). Although cluster 2 (a group that tends to vaccine hesitancy), some respondents in this same cluster (37.3%) have registered for the vaccination. This is not unusual because individuals who have made an appointment for vaccination (registered for the vaccination) will not necessarily show up or attend the appointment, as reported by most Malaysia’s newspapers <https://themalaysianreserve.com/2021/05/28/many-fail-to-show-up-for-their-vaccination-appointment/>.  This demonstrates that just because they have previously registered for the vaccination does not mean they do not support the programme. This means that something is still reasonable if we consider individuals who have registered for the vaccine to be sampled to identify the vaccine hesitancy cluster. In other words, although a small proportion of Cluster 2 respondents have registered for vaccination, their tendency not to take the vaccine (vaccine hesitancy) remains high. This will certainly not affect the hypothesis. |
| **Editor** | Use of k-means clustering requires for equal variance of all variables and the same prior probability for all k clusters. Incase variances are unequal, you can reconsider for modified k-means. | Thank you for the comments. Based on our understanding, K-Means Clustering does not require any assumptions to be met. Therefore, I just apply this algorithm which minimizes the SSE, the within cluster square error. As a result, we hope you may share your expertise in determining the variance of all variables being the same. It is beyond my knowledge. |
| **Editor** | Application PCA assumes existence of correlation between the variables, so a correlation matrix and its significance values can be added prior to PCA. | The correlation matrix table and significance values have been added as suggested by the Editor |
| **Editor** | Authors have used non-parametric tests for testing of hypothesis. Critical tests of Normality needs to be conducted pre-testing of hypothesis and application of testing techniques. | We have included the normality test schedule as recommended by the reviewer |
